# Uridine treatment protects against blood–brain barrier disruption in a rat model of Li-pilocarpine-induced status epilepticus

**DOI:** 10.3389/fnins.2025.1635247

**Published:** 2025-08-19

**Authors:** Birnur Aydin, Busra Ocalan Esmerce, Aysen Cakir, Sueda Tuncak, Cansu Koc, Mehmet Cansev, Tulin Alkan

**Affiliations:** ^1^Department of Physiology, Bursa City Hospital, Bursa, Türkiye; ^2^Department of Physiology, Faculty of Medicine, Bursa Uludag University, Bursa, Türkiye; ^3^Department of Pharmacology, Faculty of Medicine, Bursa Uludag University, Bursa, Türkiye

**Keywords:** status epilepticus, uridine, blood–brain barrier, brain edema, aquaporin-4

## Abstract

**Introduction:**

Blood–brain barrier (BBB) disruption is one of the most striking changes triggered by status epilepticus, which deserves specific attention in terms of novel treatment approaches targeting epileptogenesis. Uridine is a pyrimidine nucleoside with neuroprotective, antiepileptic and antiepileptogenic effects; however, its mechanism of action is not fully characterized. In this study, we aimed to investigate the short-term outcomes of uridine treatment on status epilepticus-induced-BBB dysfunction in an animal model.

**Methods:**

Status epilepticus was induced by lithium and pilocarpine administration in male Sprague–Dawley rats which were post-treated with intraperitoneal injection of saline or uridine (500 mg/kg b.w.; twice a day) for 2 days. Blood–brain barrier structural integrity was assessed by measuring expressions of endothelial tight junction proteins zonula occludens-1 (ZO-1) and occludin, matrix metalloproteinases (MMP-2 and MMP-9), aquaporin-4 (AQP4) water channel and its anchoring protein α1-syntrophin in hippocampal tissue 48 h after SE. Additionally, BBB permeability was determined by measuring brain edema and serum S100B levels.

**Results:**

The data showed that uridine significantly prevented the reduction in ZO-1 and α1-syntrophin protein levels and attenuated serum S100B levels, indicating protective effects on BBB integrity and AQP4 polarization. In contrast, uridine enhanced brain water content in SE-induced rats, a finding that might be a result of maintained AQP4 polarization and enhanced cytotoxic edema.

**Discussion:**

Together, our results showed for the first time that post-seizure treatment with uridine provides protection against BBB disruption in an experimental SE model; nevertheless, the long-term effects of this treatment warrant further investigation.

## 1 Introduction

Status epilepticus (SE) is a neurological emergency characterized by abnormally prolonged seizures with an overall mortality rate of 20% (Logroscino et al., 2005; Trinka and Kälviäinen, 2017). In addition to acute mortality, it triggers epileptogenesis by various mechanisms including axonal sprouting, dendritic plasticity, blood–brain barrier (BBB) disruption and neuroinflammation (Pitkänen and Lukasiuk, 2011). Terminating seizures before the onset of epileptogenic changes is crucial, although this is mostly challenging due to the drug-resistant nature of status epilepticus. Therefore, developing novel treatments to limit the long-term effects of SE becomes essential.

Blood–brain barrier damage is one of the earliest alterations detected following prolonged seizures (van Vliet et al., 2015) and can be a therapeutic target due to its substantial involvement in epileptogenesis. Excessive glutamate damages endothelial cells by elevating intracellular production of reactive oxygen radicals and causes degradation of endothelial tight junction proteins by augmenting the activity of matrix metalloproteinases (MMPs) (Sharp et al., 2005; Vazana et al., 2016; Rempe et al., 2018). Increased permeability promotes serum protein extravasation into the extracellular space, triggering neuronal hyperexcitability (Löscher and Friedman, 2020). Enhanced MMP activity is also associated with the deterioration of the dystrophin complex in the basement membrane, which in turn leads to aquaporin-4 (AQP4) depolarization (Eid et al., 2005). AQP4 is enriched in astrocyte end-feet surrounding microvessels, a condition known as polarization, and it co-localizes in the astrocyte membrane with Kir4.1 potassium channels (Guadagno and Moukhles, 2004). Loss of polarization affects K^+^ buffering and brain tissue water clearance, leading to neuronal hyperexcitability, increased seizure severity and neurodegeneration (Nagelhus et al., 2004; Eid et al., 2005; Szu and Binder, 2022). Hence, BBB disruption as a consequence of status epilepticus induces seizures, resulting in a vicious circle that must be interrupted (Löscher and Friedman, 2020).

Uridine is the major pyrimidine nucleoside in human blood circulation (Cansev, 2006). Several experimental studies have revealed the neuroprotective benefits of exogenous administration of uridine (Dobolyi et al., 2011; Cansev et al., 2013). Moreover, antiepileptic and antiepileptogenic characteristics of uridine have been demonstrated, as evidenced by its ability to reduce spike–wave discharges and neuronal damage (Zhao et al., 2008; Kovács et al., 2013). Potential mechanisms of uridine’s action in animal models of epilepsy were shown to include stimulation of GABAergic (Guarneri and Guarneri, 1983) and pyrimidinergic (Alves et al., 2017) receptors, on the other hand, its effects on non-neuronal mechanisms underlying epilepsy pathophysiology deserve further investigation. In a limited number of studies, uridine and its dinucleotide form uridine-5′-diphosphate (UDP) were shown to attenuate brain edema in experimental models of traumatic (Kabadi and Maher, 2010) and cryogenic (Yoshida et al., 1989) brain injury, respectively. In addition, evidence was provided with regard to reduced immunoglobulin G extravasation in an animal model of lipopolysaccharide-induced BBB damage by the uridine pro-drug PN401 (Saydoff et al., 2013). Based on these findings, we hypothesized that one of the mechanisms that could mediate the antiepileptogenic effect of uridine might involve maintenance of BBB integrity and aimed to determine the short-term outcomes of uridine administration in a rat model of status epilepticus. Thus, we administered rats with Li-pilocarpine to induce SE, which was followed by intraperitoneal uridine (500 mg/kg b.w.; twice a day) treatment for 48 h. BBB structural integrity was assessed by measuring expressions of endothelial tight junction proteins (ZO-1 and occludin), MMPs, AQP4 water channel, and its anchoring protein, α1-syntrophin, in the hippocampal tissue. In addition, BBB permeability was determined by measuring brain edema and serum S100B levels.

## 2 Materials and methods

### 2.1 Experimental animals

The experimental protocols were approved by the Animal Care and Use Committee of Bursa Uludag University, Bursa, Turkey (Approval ID: 2019-13/07), and were conducted in accordance with the NIH Guide for the Care and Use of Laboratory Animals. Male Sprague–Dawley rats (6–8 weeks old) were obtained from Experimental Animal Breeding and Research Center, Bursa Uludag University Medical School, Bursa, Turkey, and were kept under standard laboratory conditions (ambient temperature, 22°C; humidity, 40%) on a 12-h light/dark cycle with free access to water and food. All efforts were made to minimize the number of animals used and their suffering. The total number of experimental animals used was 115 (4 rats were excluded from the study because SE could not be induced despite the maximum dose of pilocarpine. Eight rats in the SE group and 7 rats in the SE + URI group died after SE.) The experimental groups were designed as follows (*n* = 16 per group): saline-treated sham group (SHAM), uridine-treated sham group (SHAM+URI), saline-treated control group (CTRL), uridine-treated control group (CTRL+URI), saline-treated status epilepticus group (SE), and uridine-treated status epilepticus group (SE + URI). The sham groups (SHAM and SHAM+URI) received normal saline only. The control groups (CTRL and CTRL+URI) received LiCl, methyl scopolamine, normal saline, and diazepam but did not receive pilocarpine. SE groups (SE and SE + URI) received LiCl, methyl scopolamine, pilocarpine, and diazepam. The control, sham, and SE groups were randomly subdivided into two groups based on the treatment (uridine or saline) regimen during a 48-h follow-up period.

### 2.2 Induction of status epilepticus

Status epilepticus was induced as previously described (Bankstahl et al., 2018). Lithium chloride (127 mg/kg, intraperitoneally [*ip*], CAT #L9650 Sigma-Aldrich, USA) was administered approximately 16–24 h prior to pilocarpine. Rats were then pre-treated with methyl scopolamine nitrate (1 mg/kg, *ip*, CAT#S8502 Sigma-Aldrich, USA) 30 min before pilocarpine administration to reduce the peripheral effects of pilocarpine. To induce SE, the rats received repeated *ip* injections of pilocarpine (10 mg/kg, CAT#P6503 Sigma-Aldrich, USA) every 20–30 min until the onset of continuous generalized convulsive seizures. The Racine scale was used to assess seizure severity (Racine, 1972). Status epilepticus was defined as continuous tonic–clonic seizures or intermittent stage 4 and 5 seizures without recovery of normal conscious behavior between seizures. Animals with stage 4–5 seizures were included in the study. To decrease mortality, the duration of status epilepticus was limited to the shortest period in which epileptogenic changes were observed (Klitgaard et al., 2002; Bortel et al., 2010), and diazepam (10 mg/kg, *ip,* Diazem, DEVA, Turkey) was injected 30 min after the onset of stage 4-5 seizures and repeated as needed, up to a maximum dose of 25 mg/kg. Four rats in which SE could not be induced were excluded from the study. Post-SE mortality resulted in the loss of 8 rats from the SE group and 7 rats from the SE + URI group, yielding an overall SE-induced mortality rate of 31.9%. After terminating SE, rats were randomly assigned to one of two groups: SE + URI or SE group.

**Figure 1:**
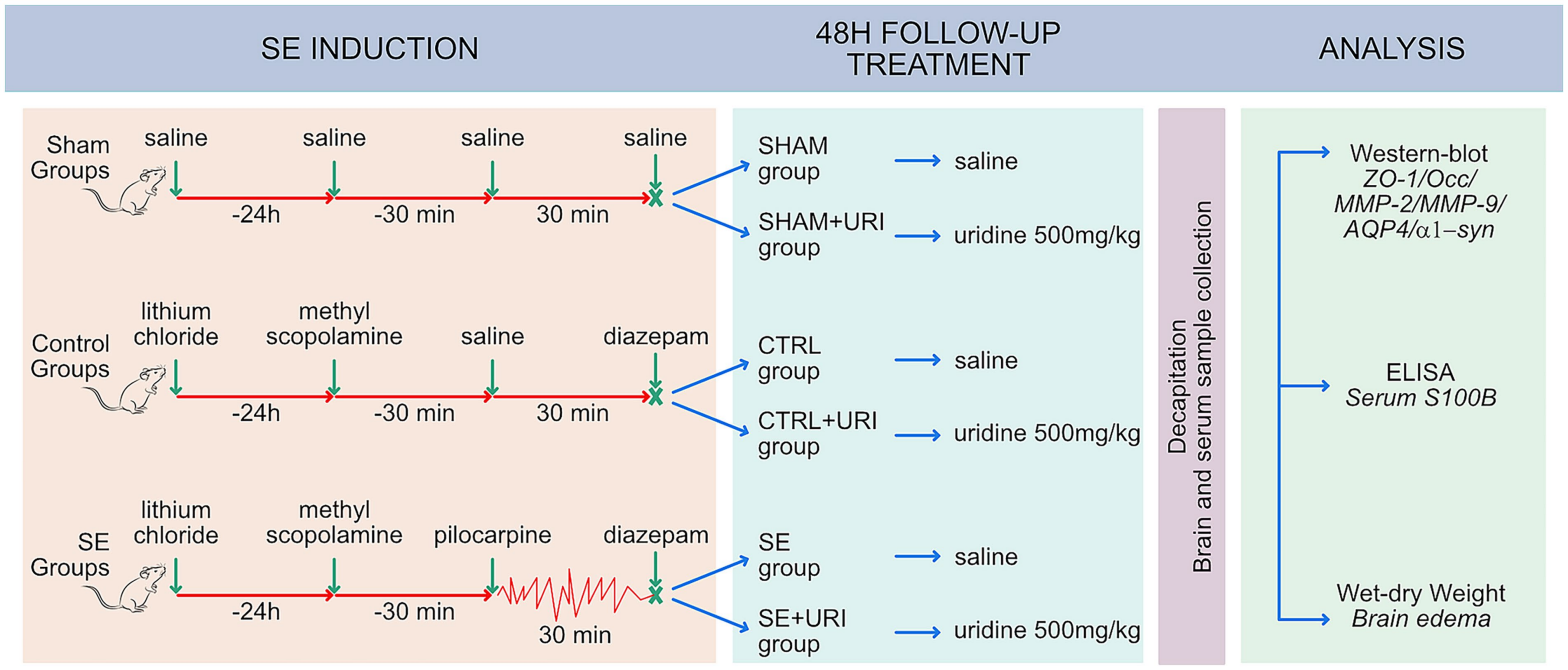
Study design. Male Sprague–Dawley rats received lithium chloride 16–24 h prior to pilocarpine. Status epilepticus was induced repetitive low-dose pilocarpine administration and SE was terminated by diazepam, 30 min after reaching stage 4–5 seizures. Rats were post-treated with intraperitoneal injection of saline or uridine (500 mg/kg b.w.; twice a day) for 2 days. Tight junction proteins (ZO-1, occludin), matrix metalloproteinases (MMP-2 and MMP-9), aquaporin-4 and α1-syntrophin in hippocampal tissue 48 h after SE were evaluated using western blot. BBB permeability was determined by measuring brain edema and serum S100B levels. AQP4, aquaporin-4; α1-syn, α1-syntrophin; MMP-2, matrix metalloproteinase-2; MMP-9, matrix metalloproteinase-9; Occ, occludin; ZO-1, zonula occludens-1; SE, status epilepticus.

The rats in the SHAM+URI, CTRL+URI, and SE + URI groups received uridine (500 mg/kg, *ip,* CAT#U3750 Sigma-Aldrich, USA) immediately after SE and twice a day for 48 h, while those in the SHAM, CTRL and SE group received an equivalent volume of saline. The selected dose of uridine (500 mg/kg) has previously been shown to exhibit neuroprotective effects in a model of hypoxic–ischemic encephalopathy (Cansev et al., 2013) and decrease absence epileptic activity (Kovács et al., 2015). Animals were kept in plexiglass cabinets during SE, and seizures were recorded using a video camera (Hikvision, Hangzhou, China). The experimental protocol is illustrated in Figure 1.

### 2.3 Western blotting

Eight animals in each group were deeply anesthetized with isoflurane and decapitated 48 h after SE induction, and their brains were removed immediately thereafter. The right hippocampus was dissected and homogenized in ten volumes of ice-cold phosphate-buffered saline (PBS, pH 7.4) and stored at −80°C for western blot analysis.

Western blot analysis was performed according to widely established protocols. Briefly, total protein was determined using the Lowry method (Lowry et al., 1951). Each sample was incubated with Laemmli buffer for 5 min at 95°C (Laemmli, 1970); then, 20 μg of proteins were loaded and separated by electrophoresis on 12% separating gel (SDS-PAGE; Mini Protean II, Bio-Rad, USA) and transferred onto polyvinylidene difluoride membrane (CAT#IPVH00010, Millipore, USA). Membranes were blocked with 5% non-fat skim milk in Tris-buffered saline containing 0.1% Tween 20 (TBST) for 1 h and incubated with primary antibodies [anti-AQP4 (1:1000, CAT#E-AB-64864 Elabscience, USA), anti-syntrophin α1 (1:1000, CAT#bs-3600R, Bioss, USA), anti-ZO-1 (1:500, CAT#sc-33725 Santa Cruz Biotechnology, USA), anti-occludin (1:1000, CAT#BS-1495R, Bioss, USA), anti-MMP-2 (1:500, CAT#sc-13595 Santa Cruz Biotechnology, USA) and anti-MMP-9 (1:750, CAT#sc-393859, Santa Cruz Biotechnology, USA)] overnight at 4°C. Following incubation, samples were washed three times for 5 min in TBST and then incubated with the corresponding horseradish peroxidase-conjugated secondary antibody (Cell Signaling Technology, USA) for 1 h at room temperature. Proteins were visualized using enhanced chemiluminescence (CAT#WBLUR0500, Millipore, USA), digital images were developed, and optical densities of proteins were analyzed using a digitized scanner (CDigit, LI-COR Biotechnology, USA). *β*-Actin was used as a loading control (1:1000, CAT#4967, Cell Signaling Technology, USA).

### 2.4 Enzyme-linked immunosorbent assay

To evaluate BBB permeability, we measured serum S100B levels, which have been proposed as a peripheral indicator of BBB damage (Marchi et al., 2004). Trunk blood was obtained after decapitation and centrifuged for 5 min at 10,000 rpm and 4°C. Serum samples were collected and stored at −80°C for analysis. Serum S100B levels were quantitated using a specific enzyme-linked immunosorbent assay (ELISA) kit (CAT #E1360Ra, Bioassay Technology Laboratory, China) by following the manufacturer’s instructions. The standard S100B curve ranged from 5–1500 pg./mL, and the data were expressed as picograms per milliliter.

### 2.5 Brain water content

We measured brain water content using the wet-dry weight method to evaluate brain edema at 48 h post-SE since brain edema peaks at 24–48 h after SE (Bankstahl et al., 2018). Eight rats in each group were euthanatized, and brains were dissected immediately following decapitation. Whole brains were weighed immediately after decapitation as well as following drying procedure in an oven for 24 h at 110°C. The percentage of water content was calculated using the following equation, as previously described (Elliott and Jasper, 1949):

$$\begin{array}{l} \%\text{Brain water content}=\\ {}[(\mathrm{wet}\;\text{weight}-\mathrm{dry}\;\text{weight})/\mathrm{wet}\;\text{weight}]\times 100. \end{array}$$

### 2.6 Statistical analysis

Statistical analysis was performed using SigmaPlot (version 12.5). The Shapiro–Wilk test was used to determine whether the data were normally distributed. Descriptive statistics were expressed as mean ± standard error of means (SEM) for quantitative data. Groups were compared using One-way analysis of variance (ANOVA) followed by *post hoc* Holm-Sidak test for pairwise comparisons. The significance level was set at *α* = 0.05.

## 3 Results

### 3.1 Effects of uridine on hippocampal endothelial tight junction protein expression and matrix metalloproteinase protein levels

**Figure 2:**
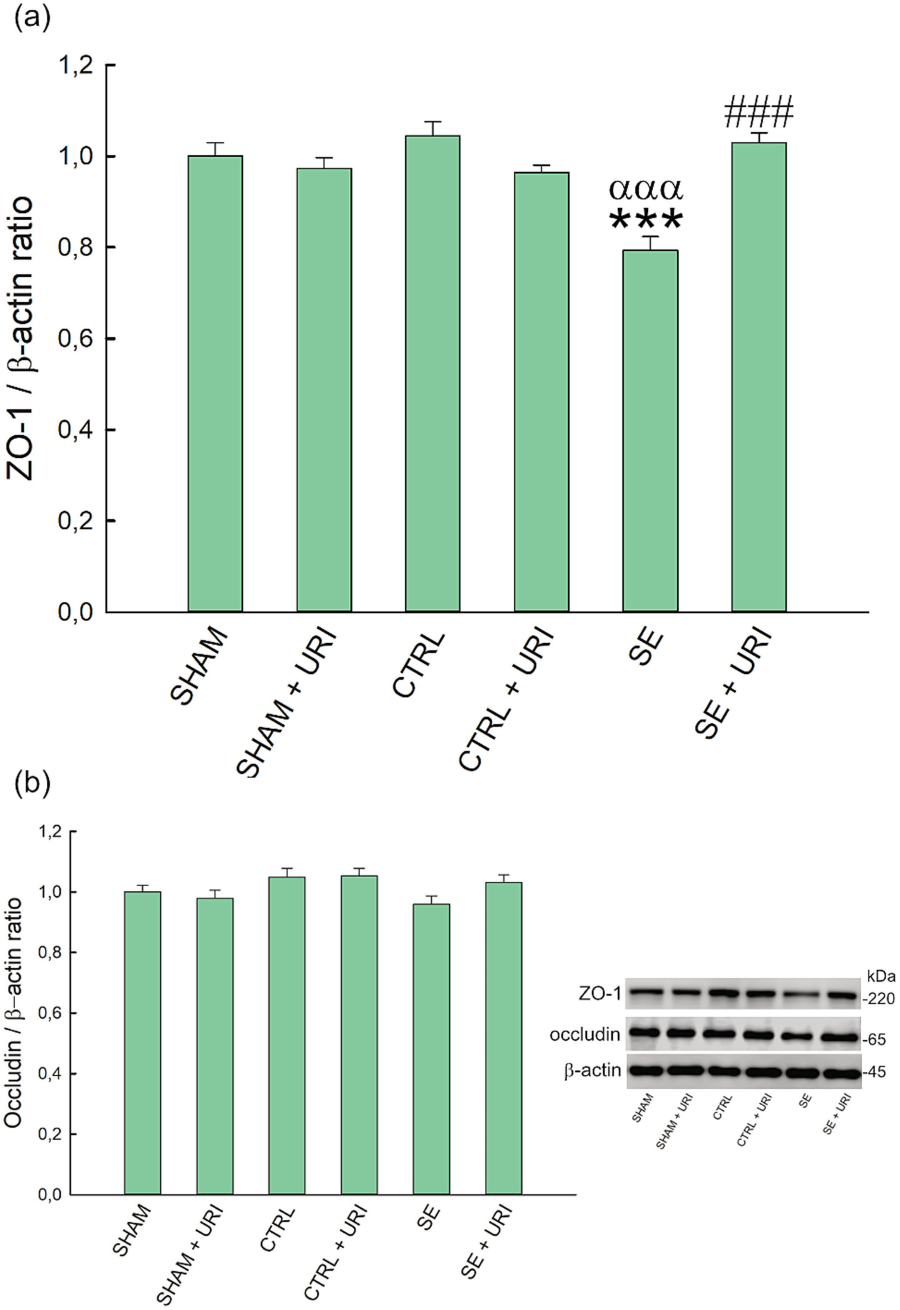
Effect of uridine treatment on expression levels of ZO-1 **(a)** and occludin **(b)** in Li-pilocarpine-induced SE. Representative western blot of related proteins. Significance was determined using one-way ANOVA followed by Holm-Sidak *post hoc* test. Data were expressed as mean ± SEM. *β*-actin was used as loading control. ***p < 0.001 vs. SHAM, ^ααα^*p* < 0.001 vs. CTRL, ^###^*p* < 0.001 vs. SE. *n* = 8 in each group. ZO-1, zonula occludens-1; SE, status epilepticus.

Zonula occludens-1 and occludin expression in the hippocampus were determined to investigate the effect of uridine on endothelial tight junctions and BBB integrity. Hippocampal ZO-1 expression was significantly lower in the SE group compared to those in the SHAM and CTRL groups (*p* < 0.001 SHAM vs. SE and *p* < 0.001 CTRL vs. SE) (Figure 2a). Uridine treatment prevented ZO-1 expression from decreasing (*p* < 0.001 SE vs. SE + URI). In contrast, hippocampal occludin expression did not differ between the groups (*p* = 0.082) (Figure 2b).

**Figure 3:**
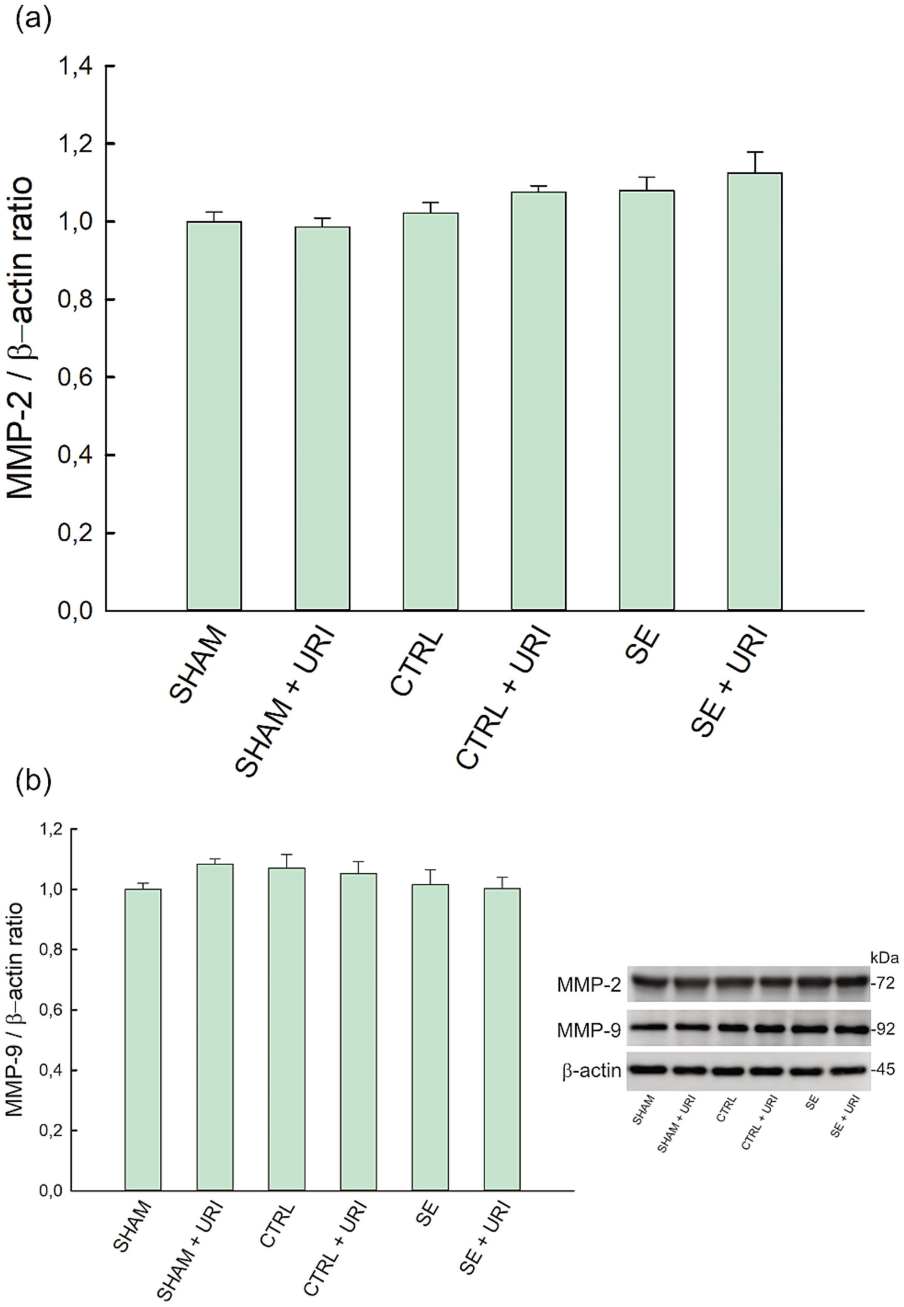
Effect of uridine treatment on matrix metalloproteinase level in Li-pilocarpine-induced SE. Expression levels of MMP-2 **(a)** and MMP-9 **(b)** and representative blots. Data were analyzed with one-way ANOVA followed by Holm-Sidak *post hoc* test and expressed as mean ± SEM. β-actin was used as loading control. *n* = 8 in each group. MMP-2, matrix metalloproteinase-2; MMP-9, matrix metalloproteinase-9.

MMP-2 and MMP-9 levels were measured using western blotting. One-way ANOVA showed a significant difference in the expression of MMP-2 (*p* = 0.024); however, *post hoc* multiple comparison test revealed no statistically significant differences between groups. No change of hippocampal MMP-9 expression was observed between analyzed groups (*p* = 0.422) (Figure 3).

### 3.2 Effects of uridine on hippocampal AQP4 and α1-syntrophin protein expression

**Figure 4:**
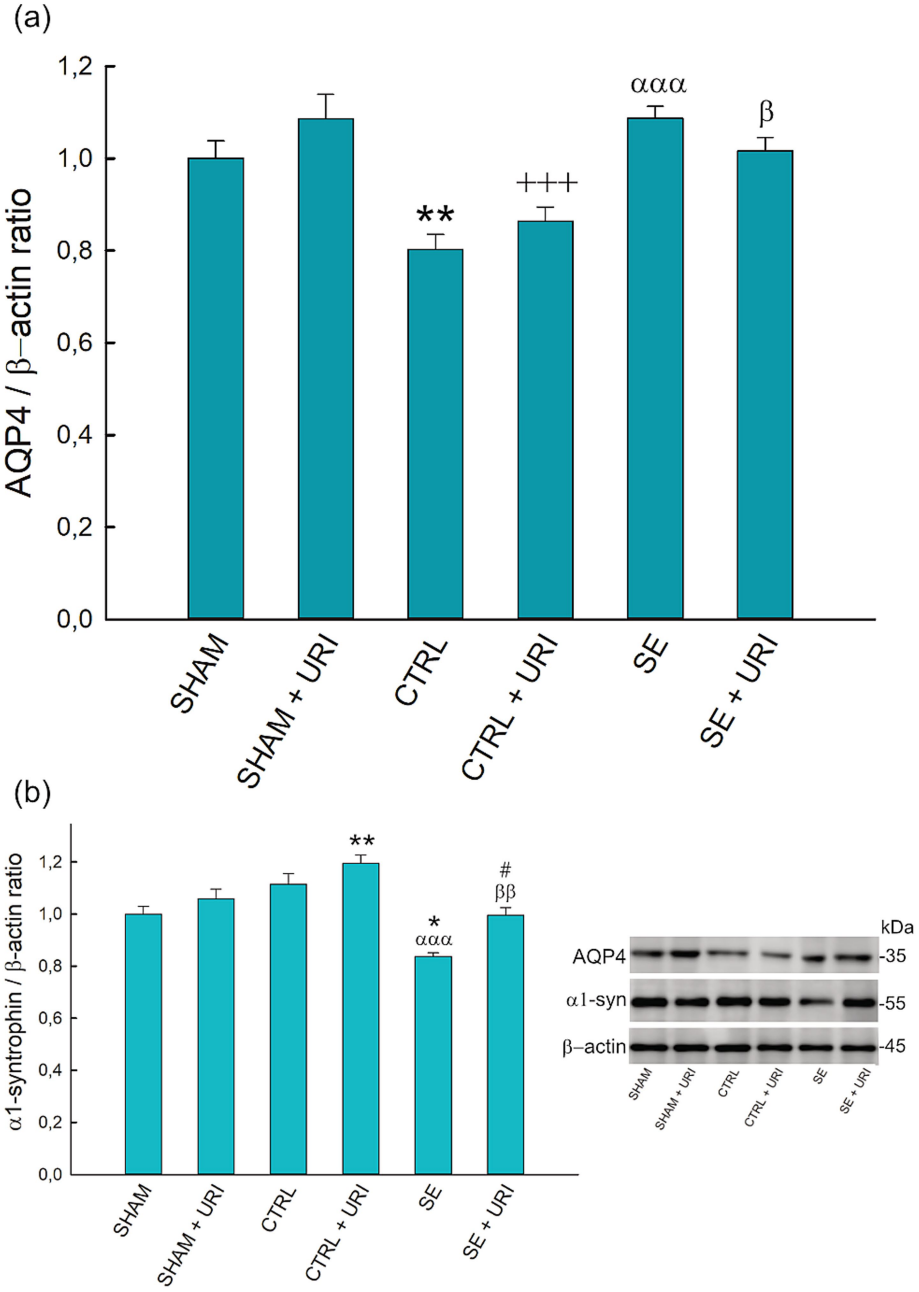
Effect of uridine treatment on AQP4 and α1-syntrophin protein expression following SE. Graphs describing relative expression of AQP4 **(a)** and α1-syntrophin **(b)** and representative blots. One-way ANOVA followed by Holm-Sidak *post hoc* test. Data were expressed as mean ± SEM. *p < 0.05, **p < 0.01 vs. SHAM, ^+++^*p* < 0.001 vs. SHAM+URI, ^ααα^*p* < 0.001 vs. CTRL, ^β^*p* < 0.05, ^ββ^*p* < 0.01 vs. CTRL+URI, ^#^*p* < 0.05 vs. SE. *n* = 8 in each group. AQP4, aquaporin-4; α1-syn, α1-syntrophin.

We analyzed AQP4 protein expression to evaluate the effect of uridine on AQP4 polarization. As shown in Figure 4a, no statistically significant difference in AQP4 protein expression was found between the SE and SE + URI groups compared to the SHAM groups. Unexpectedly, AQP4 expression was significantly decreased in both the CTRL and CTRL+URI groups that received lithium, scopolamine, and diazepam but not pilocarpine (*p* = 0.003 SHAM vs. CTRL, *p* < 0,001 SHAM+URI vs. CTRL+URI).

Next, we investigated α1-syntrophin protein expression as an anchoring protein responsible for the polarized expression of AQP4. In contrast to AQP4, α1-syntrophin protein expression was significantly lower in the SE group than that in the SHAM and CTRL groups (*p* = 0.014 and *p* < 0.001, respectively). Uridine treatment prevented the decrease in α1-syntrophin levels after SE (*p* = 0.023 SE + URI vs. SE) (Figure 4b).

### 3.3 Effects of uridine on S100B protein levels in serum

**Figure 5:**
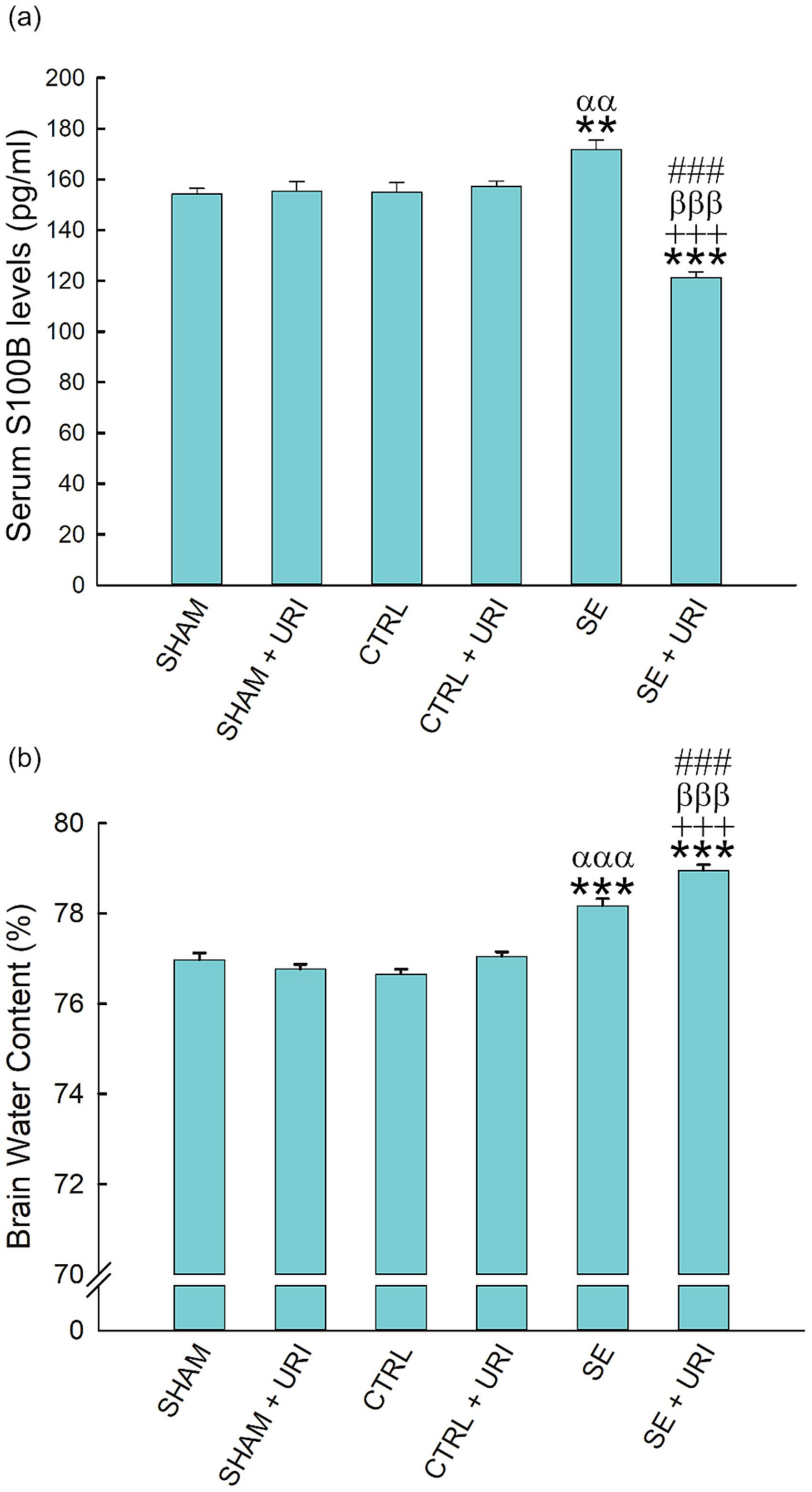
Brain edema and serum S100B levels following Li-pilocarpine-induced SE. Brain water content **(a)** was determined with wet-dry weight method. Serum S100B levels **(b)** were measured by ELISA. One-way ANOVA followed by Holm-Sidak *post hoc* test. Data were expressed as mean ± SEM. **p < 0.01, ***p < 0.001 vs. SHAM, ^+++^*p* < 0.001 vs. SHAM+URI, ^αα^*p* < 0.01, ^ααα^*p* < 0.001 vs. CTRL, ^βββ^*p* < 0.001 vs. CTRL+URI, ^###^*p* < 0.001 vs. SE. *n* = 8 in each group.

We analyzed serum S100B levels using ELISA as a peripheral marker of BBB dysfunction. Serum S100B content was elevated after SE induction at 48 h (SE vs. SHAM: 171.72 ± 3.74 vs. 154.26 ± 2.20 pg./mL; SE vs. CTRL: 171.72 ± 3.74 vs. 155.00 ± 3.70 pg./mL, *p* < 0.001). Uridine treatment significantly decreased S100B levels (SE vs. SE + URI: 171.72 ± 3.74 vs. 121.25 ± 2.43 pg./mL, *p* < 0.001) (Figure 5a).

### 3.4 Effects of uridine treatment on brain Edema

The brain water content in the SHAM group was 76.97 ± 0.15%. As shown in Figure 5b, at 48 h post-SE, brain water content was significantly elevated in the SE group (SE vs. SHAM: 78.17 ± 0.16 vs. 76.97 ± 0.15, *p* < 0.001; SE vs. CTRL: 78.17 ± 0.16 vs. 76.66 ± 0.11, *p* < 0.001). Contrary to expectations, brain water content was further enhanced in the SE + URI group compared to that in the SE group (SE vs. SE + URI: 78.17 ± 0.16 vs. 78.95 ± 0.13, *p* < 0.001) (*n* = 8 per group).

## 4 Discussion

In the present study, we investigated the effect of acute and short-term uridine treatment on status epilepticus-induced BBB dysfunction. Using the Li-pilocarpine model, we treated rats with uridine after SE and assessed BBB integrity and leakage. We found for the first time that uridine, administered immediately after SE, significantly prevented ZO-1 and α1-syntrophin protein degradation in the hippocampus and effectively decreased serum S100B levels. These data suggest that uridine may provide protection by maintaining BBB structural integrity and preventing the loss of AQP4 polarization following SE. In contrast, uridine increased brain edema in SE-induced rats, a finding that might be associated with greater AQP4 polarization and enhanced cytotoxic edema.

Chemoconvulsant-induced SE models have been widely used in epilepsy research, with the Li-pilocarpine model being one of the most common. The Li-pilocarpine model was utilized in this study due to its well-documented capacity to recapitulate core features of human temporal lobe epilepsy (TLE), particularly its characteristic histopathological progression. This model reliably induces neuronal necrosis within 24 h in brain regions critically involved in human TLE, including the hippocampus, amygdala, thalamus, piriform and entorhinal cortex, substantia nigra, and cerebral cortex (Covolan and Mello, 2000). Furthermore, it mirrors the glutamate excitotoxicity observed in human epilepsy, manifesting as dendritic swelling, vacuolar degeneration in neuronal somata, and astroglial dilatation (Clifford et al., 1987). Crucially, the acute SE triggered by systemic pilocarpine administration, followed by the development of chronic spontaneous recurrent seizures, establishes this model as one of the most clinically relevant for studying TLE pathogenesis and pathology (Brandt et al., 2016). Our experimental focus on the acute phase (48 h post-SE) aligns with this model’s strength in producing early, observable lesions (Lévesque et al., 2016) and is supported by literature demonstrating significant blood–brain barrier disruption (decreased tight junction proteins) and peak brain edema within this timeframe (Bankstahl et al., 2018; Rempe et al., 2018). Repeated low-dose pilocarpine was administered to lithium-pretreated rats to reduce mortality, based on previous reports (Glien et al., 2001). SE was induced in 92.16% of the animals; however, the mortality rate was greater than previously reported (Glien et al., 2001).

Blood–brain barrier disruption has been recognized as a causal pathogenic component of epilepsy in experimental and clinical studies, and this dysfunction is caused by failures in many cellular components involved in the barrier structure (Abbott et al., 2010). Endothelial tight junction proteins are essential for the barrier function, and expressions of tight junction proteins are altered by status epilepticus through enhanced eNOS levels and MMP activity (Kim et al., 2015; Rempe et al., 2018). Michalak et al. demonstrated a reduction in continuous staining for ZO-1 in hippocampal endothelial cells at the time point 24 h after SE, while western blotting did not support this reduction (Michalak et al., 2013). In accordance with the findings Rempe et al. demonstrated (Rempe et al., 2018), we observed a reduction in ZO-1 expression in western blotting. In contrast, we did not find a significant difference between the intervention groups on occludin levels, although occludin protein expression tended to decrease after SE. Due to the heterogeneity in the course of cellular damage in different chemoconvulsant-induced SE models, measurements at distinct time points may have led to discrepancies between studies. Studies reported a strong relationship between SE and increased MMP activity, which is associated with decreased levels of tight junction proteins and remodeling of the extracellular matrix (Mizoguchi and Yamada, 2013; Dubey et al., 2017). Rempe et al. showed that the protein expression and activities of MMP-2 and MMP-9 were enhanced in brain endothelial cells at 48 h after SE (Rempe et al., 2018). In contrast, Gorter et al. reported that MMP-3 upregulation was restricted to the entorhinal cortex in the acute phase (1 day post-SE). MMP-2 increased in both the hippocampal CA3 region and entorhinal cortex during the latent phase, while MMP-9 upregulation occurred exclusively in the CA3 region during the chronic phase; however, this study assessed only gene expression, not enzymatic activity (Gorter et al., 2007). In another study, only MMP-3 mRNA expression was upregulated in the hippocampus on days 1 and 3 post-seizure, while MMP-2 and MMP-9 did not change (Wan et al., 2022). These discrepancies highlight that MMP level alterations vary considerably across distinct brain regions and cell types. Differences in MMP activity can also occur independently of changes in mRNA or protein expression. Consequently, methodological variations and biological complexity contribute to the observed differences between studies. Although we found no statistical difference in MMP-2 and -9 protein expressions, decreased ZO-1 protein levels may imply increased MMP activity. In addition, uridine administration alleviated the decrease in ZO-1 expression following SE, restoring it to levels comparable to those observed in the sham and control groups. Previous studies have reported improved intestinal ZO-1 and occludin expressions following uridine administration (Perez-Pardo et al., 2017; Xie et al., 2019). To the best of our knowledge, this is the first study to demonstrate the protective effect of uridine on brain endothelial ZO-1 expression in an experimental model of brain injury.

AQP4, the principal water channel in the brain and spinal cord, provides bilateral water transport between the extracellular space and the blood circulation/cerebrospinal fluid and plays a fundamental role in regulating interstitial osmolality and extracellular fluid volume (Nagelhus et al., 2004). Changes in AQP4 expression have been demonstrated to contribute to epilepsy; however, previous studies evaluating AQP4 expression have provided inconsistent findings about whether its levels in epilepsy are increased, decreased, or unchanged (Eid et al., 2005; Kim et al., 2010; Hubbard et al., 2016). Although total AQP4 expression was augmented in patients with mesial temporal lobe epilepsy, perivascular AQP4 localization was decreased by 44% (Eid et al., 2005). In the same study, perivascular dystrophin staining disappeared similarly to AQP4, suggesting that the degradation of the dystrophin complex is responsible for the loss of AQP4 polarization (Eid et al., 2005). In particular, the decrease in α1-syntrophin, a component of the dystrophin complex, was accompanied by a reduction in perivascular AQP4 in the kainic acid-induced SE model (Alvestad et al., 2013). These findings are supported by a research utilizing α1-syntrophin knockout mice, which revealed a 79% decrease in perivascular AQP4 expression in the spinal cord and a 94% decrease in the neocortex, but no decrease in other astrocyte membrane domains (Amiry-Moghaddam et al., 2003; Hoddevik et al., 2017). Therefore, α1-syntrophin was considered a major anchoring protein for AQP4 polarization (Hoddevik et al., 2017). Consistent with the literature, 48 h after SE, we observed a decrease in α1-syntrophin expression but not in total AQP4, indicating disrupted perivascular localization. Administration of uridine increased the levels of α1-syntrophin in SE-induced rats, normalizing it to sham group levels. We also observed elevated alpha-syntrophin levels in the uridine-treated control group compared to the sham group. This increase could represent a compensatory mechanism protecting AQP4 polarization in response to decreased AQP4 levels, although this hypothesis requires further verification. Taken together, these results suggest that uridine may prevent the loss of perivascular AQP4 expression. Polarized expression of AQP4 is important for the brain waste and K^+^ clearance, neuronal excitability and long-term potentiation (Szu and Binder, 2022). Potassium clearance was shown to be impaired in homozygote AQP4 knockout animals, in which seizure severity was increased (Binder et al., 2006). In addition to seizure severity, mislocalization of AQP4 leads to accumulation of waste material and extracellular hyperphosphorylated tau protein, resulting in enhanced neurodegeneration (Ishida et al., 2022). In the present study, we investigated the short-term effects of uridine on AQP4 dysregulation following SE, and future studies are warranted to identify its long-term consequences within the context of seizure severity, hippocampal sclerosis, and cognitive decline.

An unexpected observation was the decreased AQP4 expression in the control groups receiving either saline or uridine compared with the respective sham groups. As previous studies investigating AQP4 expression compared the Li-pilocarpine-induced SE group with either sham or control groups, little is known regarding the comparison between sham and control groups. The difference between the control and sham groups was that rats in the control group received lithium, scopolamine, and diazepam, whereas those in the sham group did not. Therefore, the decreased AQP4 expression in the control groups vs. the respective sham groups may be associated with the administration of lithium and/or diazepam. Taler et al. (2020) showed that lithium decreased AQP4 levels in unstressed rats but not in chronic stress-induced rats; however, no observation has been reported regarding diazepam. Since these two drugs are commonly used to treat a variety of neuropathologies, there is a need for further research regarding how lithium or diazepam affects the expression of AQP4.

Several clinical and experimental studies investigated S100B’s potential as a biomarker and its prognostic value in epilepsy since it indicates astroglial damage (Freund et al., 2015; Vizuete et al., 2017; Bulduk et al., 2019). In good accord, it has been reported that serum S100B levels were increased in epilepsy patients (Liang et al., 2019) and after SE (Hanin et al., 2022). However, Marchi et al. showed that BBB dysfunction was required for the enhanced serum S100B level (Marchi et al., 2004). Their study showed an increased serum S100B level in the absence of neuronal damage in a BBB disruption model. No differences were observed in serum levels of S100B in the absence of BBB damage, even if neuronal damage was evident. Therefore, the authors claimed that the measurement of S100B in serum might be more valuable in detecting BBB disruption than neuronal damage. In the present study, we investigated the levels of S100B in serum as an indicator of BBB disruption and astrocytic activation. We observed, in line with the previous studies (Bulduk et al., 2019; Hanin et al., 2022), that serum S100B levels were increased in SE-induced rats. Uridine administration after SE significantly reduced serum S100B levels. Therefore, our data on serum S100B levels suggest protection of BBB integrity by uridine, which corresponds with the finding that uridine enhanced ZO-1 levels.

We observed that brain edema was enhanced at 48 h post-SE, which was further increased by uridine treatment. This increase was an unexpected result as it appears to contrast with observations of the protective effect of uridine on BBB structural integrity and permeability. Uridine and UDP have been shown to reduce brain edema in a limited number of studies, which inspired us during the course of developing our research hypotheses. These experiments were conducted on different animal models using varying treatment doses and administration routes. Kabadi et al. demonstrated that uridine administered at a dose of 32 mg/kg alone and in combination with 200 mg/kg melatonin decreased brain water content in the striatum 48 h after traumatic brain injury (Kabadi and Maher, 2010). In this study, the wet weight/dry weight method was used, but the type of edema (vasogenic or cytotoxic) was not determined. The enhancement in the brain edema we observed after uridine treatment might be due to cytotoxic or vasogenic edema. Brain edema after SE occurs in two phases depending on time; cytotoxic edema starts immediately after SE and lasts for several hours, while the onset of vasogenic edema takes hours due to increased BBB permeability (Itoh et al., 2015; Bankstahl et al., 2018). In the kainic acid-induced SE model, cytotoxic edema was observed at 3 h post-SE and returned to normal at 48 h, according to diffusion-weighted magnetic resonance imaging research (Righini et al., 1994). On the other hand, another study reported that cytotoxic edema was still evident after 48 h (Itoh et al., 2015). One possible explanation for the increased brain edema is that uridine may have enhanced brain water content after SE by preserving perivascular AQP4 localization. This hypothesis is further supported by the fact that AQP4 polarization plays a dual role in brain edema: Polarized expression is associated with both increased water transport to brain tissue in cytotoxic brain edema and resolution of vasogenic edema (Papadopoulos et al., 2004; Stokum et al., 2014). Therefore, our data suggest that the enhanced edema formation following uridine is associated with cytotoxic edema due to AQP4 polarization at the time of analysis (48 h post-SE). A longer follow-up period for SE-induced rats treated with uridine may reveal the resolution of edema because vasogenic edema dominates late-term edema formation.

In summary, we showed that uridine treatment maintained BBB integrity, as suggested by enhanced ZO-1 expression, reduced serum S100B levels and enhanced AQP4 polarization in rats with Li-pilocarpine-induced SE. In contrast, enhanced brain edema following SE was further increased by uridine, a finding that might be associated with AQP4 polarization, which results in enhanced cytotoxic edema. The major limitation of this study was the absence of histopathological findings to confirm AQP4 polarization and tight junction protein expression. In addition, electroencephalography could have been performed, but we avoided it for the purpose of not causing additional damage to the brain. Despite its limitations, the evidence from this study suggests that uridine may be used as an add-on therapy for diseases that accompany BBB dysfunction. Nevertheless, further studies are required to investigate the long-term effects of uridine on epileptogenesis.

## Data Availability

The raw data supporting the conclusions of this article will be made available by the authors, without undue reservation.
